# Current research status of tumor cell biomarker detection

**DOI:** 10.1038/s41378-023-00581-5

**Published:** 2023-10-05

**Authors:** Liying Jiang, Xinyi Lin, Fenghua Chen, Xiaoyun Qin, Yanxia Yan, Linjiao Ren, Hongyu Yu, Lingqian Chang, Yang Wang

**Affiliations:** 1 School of Electrical and Information Engineering, Zhengzhou University of Light Industry, Zhengzhou, 450002 China; 2 Academy for Quantum Science and Technology, Zhengzhou University of Light Industry, Zhengzhou, 450002 China; 3 Department of Mechanical and Aerospace Engineering, The Hong Kong University of Science and Technology, Clear Water Bay, Kowloon, Hong Kong SAR, China; 4 key Laboratory of Biomechanics and Mechanobiology (Beihang University), Ministry of Education, Beijing Advanced Innovation Center for Biomedical Engineering, School of Biological Science and Medical Engineering, Beihang University, Beijing, 100083 China; 5 School of Engineering Medicine, Beihang University, Beijing, 100083 China

**Keywords:** Biosensors, Sensors

## Abstract

With the annual increases in the morbidity and mortality rates of tumors, the use of biomarkers for early diagnosis and real-time monitoring of tumor cells is of great importance. Biomarkers used for tumor cell detection in body fluids include circulating tumor cells, nucleic acids, protein markers, and extracellular vesicles. Among them, circulating tumor cells, circulating tumor DNA, and exosomes have high potential for the prediction, diagnosis, and prognosis of tumor diseases due to the large amount of valuable information on tumor characteristics and evolution; in addition, in situ monitoring of telomerase and miRNA in living cells has been the topic of extensive research to understand tumor development in real time. Various techniques, such as enzyme-linked immunosorbent assays, immunoblotting, and mass spectrometry, have been widely used for the detection of these markers. Among them, the detection of tumor cell markers in body fluids based on electrochemical biosensors and fluorescence signal analysis is highly preferred because of its high sensitivity, rapid detection and portable operation. Herein, we summarize recent research progress in the detection of tumor cell biomarkers in body fluids using electrochemical and fluorescence biosensors, outline the current research status of in situ fluorescence monitoring and the analysis of tumor markers in living cells, and discuss the technical challenges for their practical clinical application to provide a reference for the development of new tumor marker detection methods.

## Introduction

While tumors are not heritable, the genetic changes contributing to tumor development can be inherited; over time, cells are genetically altered by tumor-causing factors and lose their normal growth regulation mechanisms, resulting in abnormal proliferation. Broadly, tumors can be divided into benign tumors and malignant tumors. Since benign tumors grow slowly, have clear boundaries with surrounding tissues, and do not metastasize, they are not harmful to human health. However, malignant tumors can metastasize and grow rapidly and produce harmful substances, thus posing a serious threat to human health^1,2^. The essence of a tumor is tumor cells, which are the main component of the tumor. In the process of transformation from normal cells to tumor cells, specific proteins or small molecules appear on the surface of these cells or in the serum, and screening for proteins or small molecules that are highly expressed in tumor cells as markers for tumor diagnosis is beneficial for the early detection and treatment of tumors^3,4^.

The current methods for the early diagnosis of tumor cells are imaging^5^, tissue biopsy^6^, and liquid biopsy^7^. Due to the limitations of imaging for the examination of tiny tumor cells, tissue biopsy requires a minimally invasive approach to extract cells from tumor tissue for pathological examination. In contrast, liquid biopsy detects tumors by examining biomarkers circulating in body fluids^8^, and these biomarkers include circulating tumor cells (CTCs), proteins, vesicles, circulating tumor DNA (ctDNA), and exosomes^9–12^. Among the tumor cell biomarkers commonly used for in vitro assays, CTCs are cells that are shed and enter the peripheral blood during tumor cell transformation and tumor progression, which implies that the tumor cells are about to metastasize^13^. Additionally, CTCs are found in many cancer types, such as breast, prostate, lung, and colorectal cancer. Therefore, they are considered the most promising tumor markers^14–17^. ctDNA is a cell-free DNA released into the blood by tumor cells after necrosis and apoptosis that can be actively secreted by tumor cells. The detection of ctDNA in blood can facilitate the early diagnosis of esophageal cancer^18^, colorectal cancer^19^, etc. Exosomes are small membrane vesicles containing RNA, DNA, and proteins released by cells^20^. The detection of exosome levels in body fluids such as urine, tears, and blood can aid in the early diagnosis of tumors, especially in patients with ovarian^21^, breast^22^, and pancreatic^23^ cancers who show significantly elevated exosome concentrations in the body circulation^24^. In addition, to enable more sensitive monitoring of tumor cells in real-time, some molecules such as nucleic acids and plasmids within living cells have been detected and tracked in situ in recent years. Among them, microRNAs (miRNAs) are a class of endogenous noncoding single-stranded short molecular RNAs whose abnormal expression can be used to assess the pathological status of different tumor cells^25^. Telomerase is a ribonucleoprotein, and telomerase activity is upregulated or reactivated in most human tumor cells but is mostly inhibited in normal somatic cells^26,27^.

**Fig. 1:**
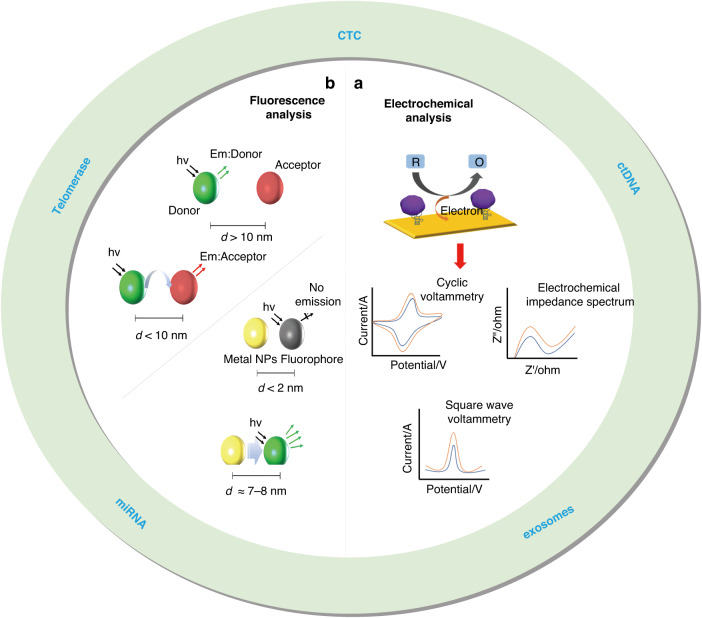
**Major biomarkers of tumor cells in liquid biopsy samples and their detection methods.** **a** Detection and analysis mechanism of electrochemical sensors^34^. Copyright 2023 MDPI. **b** Fluorescence analysis mechanism based on FRET and MEF^71^. Copyright 2021 MDPI

Many biosensors based on different signal amplification strategies have been developed in recent years for the detection of tumor cell biomarkers. Among them, electrochemical sensors have made good progress in the field of tumor marker detection because of their high sensitivity and their ability to provide relatively simple and rapid detection procedures^28^. Fluorescence analysis is also a very promising tool for intra- and extracellular tumor marker detection due to the simplicity and sensitivity of the method, but a major challenge for the in situ detection of intracellular markers in living cells using fluorescence methods is enabling the smooth passage of external biomolecules through cell membranes^29^, which is currently done mainly by penetrating cell membranes with specific nanomaterials^30^ or by using nanodevices for molecular delivery through physical methods (e.g., micro/nanoinjection^31^, electroporation^32^, etc.). This paper highlights recent methods for their detection and analysis using electrochemical/fluorescence sensing according to different tumor markers inside and outside the cells (Fig. 1). The potential role of different assays in early tumor diagnosis and the challenges they still face are also discussed. Overall, this paper summarizes the current status of the analysis of different tumor cell biomarkers and provides important concerns and insights for further research in the future.

## Electrochemical sensing

Electrochemical sensors use biomolecules such as enzymes, antibodies, and nucleic acid aptamers as recognition elements and electrochemical electrodes as conversion elements. The signal changes caused by the interaction between the biomolecules on the electrode surface and the target detector are output in the form of current or voltage using an electrochemical workstation^33–35^.

### Electrochemical sensing of tumor cell markers

Due to the advantages of high selectivity, high sensitivity, simple equipment, and low price, electrochemical sensors have been widely used for the detection of extracellular tumor markers^4,36–38^. The following sections summarize recent advances in the detection of CTCs, ctDNA, and exosomes in body fluids using electrochemical sensors.

#### Circulating tumor cells

**Fig. 2:**
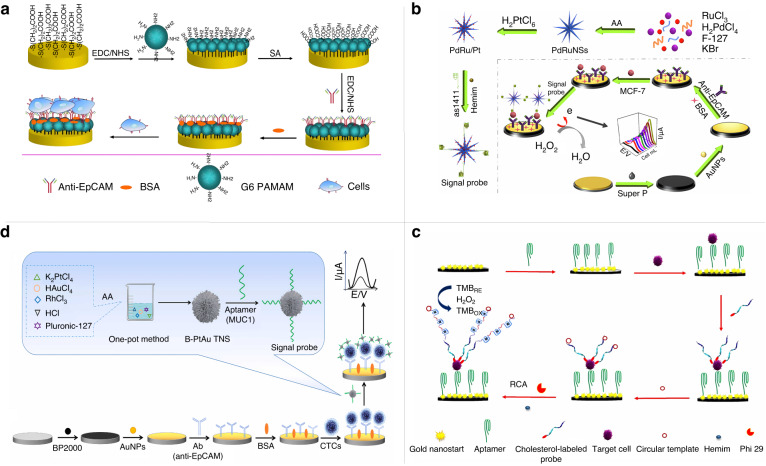
**Electrochemical sensor for detecting CTCs.** **a** A label-free electrochemical immunosensor for the detection of EpCAM overexpression in hepatocellular carcinoma cell lines^42^. Copyright 2019 MDPI. **b** Enzyme-free sandwich-type electrochemical sensor for detection of CTCs^43^, Copyright 2021 Elsevier. **c** Electrochemical aptamer sensor based on a dual-signal amplification strategy for the detection of CTCs^44^. Copyright 2021 Elsevier. **d** Electrochemical sensor based on dual recognition of anti-EpCAM antibody and anti-MUC1 aptamer^46^. Copyright 2021 Elsevier

Due to the low abundance of CTCs in peripheral blood, enrichment is usually performed based on physical characteristics such as cell size and density. This physical enrichment is simple and rapid, but the physical properties of CTCs and leukocytes are similar, which can easily lead to low purity^39,40^. Therefore, the use of protein markers overexpressed on the surface of CTCs for biological enrichment is gaining attention^41^. For example, epithelial cell adhesion molecule (EpCAM) is a transmembrane glycoprotein closely related to cancer metastasis, and the recognition of CTCs usually depends on the presence of EpCAM on the tumor cell membrane. Xu et al. developed a label-free electrochemical immunosensor for the detection of EpCAM expressed in a HepG2 hepatoma cell line with a detection limit of 2.1 × 10^3^ cells/mL (Fig. 2a)^42^. Zhou et al. first synthesized platinum nanoparticle-decorated hyperbranched PdRu nanospine (PdRu/Pt) hierarchical structures to detect CTCs with the assistance of DNAzymes^43^. As shown in Fig. 2b, the basic electrode was modified to capture CTCs specifically with EpCAM antibody. The hemin/G-quadruplex DNAzyme was integrated with PdRu/Pt as the signal probe, and the signal probe was able to recognize CTCs, forming a sandwich structure and enabling the detection of electrical signals through the catalysis of H_2_O_2_. To explore the stability of the sensor, the current response detected after storage for 21 days was maintained at the original level (98.1%). Although the stability of the immunoassay based on antigen-antibody binding is good, its specificity and sensitivity need to be improved. Electrochemical sensors based on nucleic acid aptamers effectively solve this problem. Hashkavayi et al. developed an EpCAM aptamer-based electrochemical sensor^44^. As shown in Fig. 2c, the sensor uses gold nanostar material-modified electrodes and achieves the highly sensitive detection of CTCs using a dual-signal amplification assay involving both rolling circle amplification (RCA) and the catalytic activity of hemin/G-quadruplex. This sensor is capable of the sensitive analysis of target cancer cells in the range of 5 to 10^7^ cells/mL with a detection limit of 1 cell/mL. Liu et al. used anti-EpCAM magnetic nanobeads as a capture probe and a black phosphorus-gold nanocomposite-modified aptamer as a signal probe for the efficient capture and sensitive detection of CTCs in human blood^45^. The combination of the aptamer probe and immunomagnetic separation technology, followed by the reaction of phosphite and phosphate ions on the aptamer with molybdate to generate electrochemical currents to achieve dual-signal amplification, achieved a detection limit of 2 cells/mL. The usefulness of the sensor was also validated by the sensitive detection of breast cancer cells in human blood, so this sensor provides an ultrasensitive strategy for the clinical detection of CTCs for cancer diagnosis. In addition to EpCAM, mucin 1 (MUC1) is overexpressed on CTCs from patients with metastatic lung, pancreatic, and colon cancers. Cai et al. developed an electrochemical sensor based on dual recognition of anti-EpCAM antibodies and anti-MUC1 aptamers^46^. As shown in Fig. 2d, the gold electrode surface was modified using Cabot carbon black (BP2000), and the BP2000/AuNP composites assembled more complexes with anti-EpCAM antibodies as capture probes and anti-MUC1 aptamer-linked branched PtAuRh trimetallic nanospheres as signal probes. This strategy can only detect CTCs expressing both EpCAM and MUC1, effectively improving the sensitivity of the sensor with a detection range of 5–1 × 10^6^ cells/mL and a detection limit as low as 1 cell/mL. Moreover, the proposed cell sensor can detect CTCs in clinical blood samples and is expected to be a promising platform for tumor diagnosis and metastasis detection.

#### Circulating tumor DNA

**Fig. 3:**
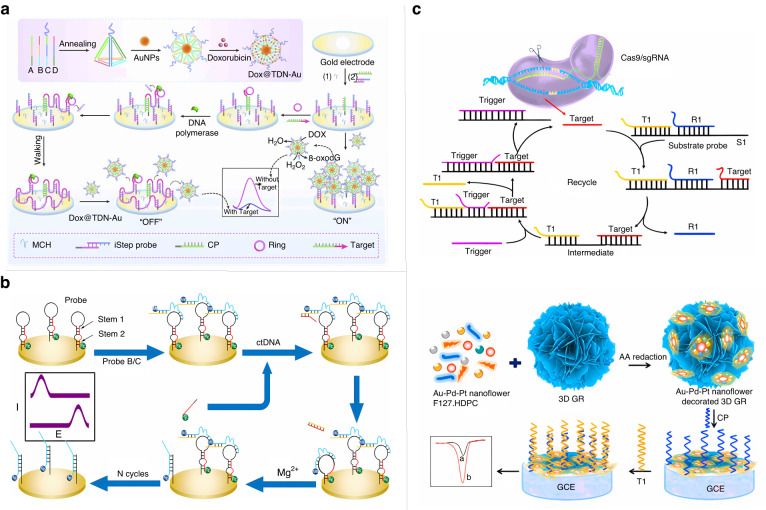
**Electrochemical sensor for detection of ctDNA.** **a** RCA-driven DNA walker-based enzyme-free electrochemical sensor for ultrasensitive analysis of ctDNA^53^. Copyright 2019 Elsevier. **b** Proportional electrochemical sensor for ctDNA based on circular amplification of DNAzyme^54^. Copyright 2022 American Chemical Society. **c** 3D GR/AuPtPd nanoflower-based system combined with CRISPR/Cas9-triggered ESDR system for electrochemical detection of ctDNA60. Copyright 2020 Elsevier

The detection of ctDNA mainly relies on traditional gene sequencing^47^ and polymerase chain reaction (PCR) methods^48^. These techniques are cumbersome and have limited the further application of ctDNA-based strategies in clinical testing to some extent^49^. Electrochemical biosensors with high sensitivity and low detection cost are gradually being developed for ctDNA detection. For example, Huang et al. proposed a novel hybridization chain reaction (HCR) system for electrochemical sensors based on nonlinear amplification involving three different types of dumbbell-shaped DNA probes that enable the formation of nested DNA structures^50^. The formation of nested DNA nanostructures helps to shorten the distance between the gold electrode surface and the reaction substrate in the electrochemical system, thereby facilitating the charge transfer process. The detection limit of ctDNA using this hybrid-stranded, nested DNA structure reached 3 pM. Compared with other DNA strand replacement amplification methods, DNA Walker has the advantages of programmability and precise base pairing and is widely used in the field of biosensors^51,52^. Li et al. designed an RCA-driven DNA Walker as an enzyme-free electrochemical sensor for the ultrasensitive analysis of ctDNA (Fig. 3a)^53^. The electrode consists of a capture probe and an iStep probe, which can specifically recognize the target DNA, and the RCA is triggered immediately when the target sequence is detected. The prominent region of the iStep probe is continuously replaced by the strand replacement reaction to form more stable double-stranded DNA. The RCA is triggered as soon as the target sequence is detected. This strategy showed good specificity and reproducibility in real sample analysis with a detection limit of 0.29 fM. Chai et al. developed a ratiometric electrochemical sensor for ctDNA detection to improve sensitivity while eliminating external influences and spurious signals^54^. As shown in Fig. 3b, in the original state, the electron mediator Fc signal is “on” and MB is “off”. Upon recognition of ctDNA, the closed DNAzyme strand at the top of the DNA structure at the electrode interface is activated, and the subsequently broken strand contains a target sequence that sequentially initiates the next reaction cycle, leading to a switch between the two electrochemical signals. Amplification by the cyclic activation of the DNAzyme has a detection limit as low as 25 aM. While a single enzyme or DNA strand substitution can be used to amplify the detection signal of the sensor, the combination of nanomaterials and electrochemical sensors can also be used to effectively improve the sensitivity of the detection; for example, Peng et al. constructed an electrochemical sensing platform for ctDNA detection using urchin-like gold nanocrystal-multiple graphene aerogel^55^. Chen et al. developed methylene blue-labeled gold magnetic nanoparticle DNA probes for electrochemical analysis of ctDNA in human blood^56^. In addition, MXenes are widely used in the compounding of semiconductor materials to improve the photoelectron lifetime of semiconductor materials because their surfaces not only react with semiconductors in a strong interfacial chemical reaction but also provide active sites for the loading of nanomaterials and metal ions^57,58^. This material shows promise for applications in photoelectrochemical biosensors. Meng et al. prepared the first ZnSe nanodisk:Ti_3_C_2_ MXene complex combined with a toehold-mediated strand displacement reaction for the electrochemical detection of ctDNA^59^. The preparation of this new MXene-based composite opens a new avenue for research on photoelectrochemical biosensors. In recent years, clustered regularly interspaced short palindromic repeats (CRISPR) and CRISPR-associated nuclease have been topics of intense interest in the field of genetic engineering but are also being employed as new tools in the field of nucleic acid precision detection. Chen et al. constructed a CRISPR/Cas9-triggered entropy-driven strand displacement reaction system based on 3D graphene/AuPtPd nanoflowers for electrochemical detection of ctDNA^60^. As shown in Fig. 3c, based on the high specific surface area of 3D graphene/AuPtPd nanoflowers and by combining the advantages of site-specific cleavage of Cas9/sgRNA and the fast amplification kinetics of ESDR, this electrochemical biosensor was used for the multiplex detection of ctDNA in human serum doped with different concentrations under optimal experimental conditions with recoveries ranging from 91.75% to 111.5%. The sensor offers a new way to effectively detect ctDNA and shows great potential for clinical and diagnostic applications.

#### Exosomes

**Fig. 4:**
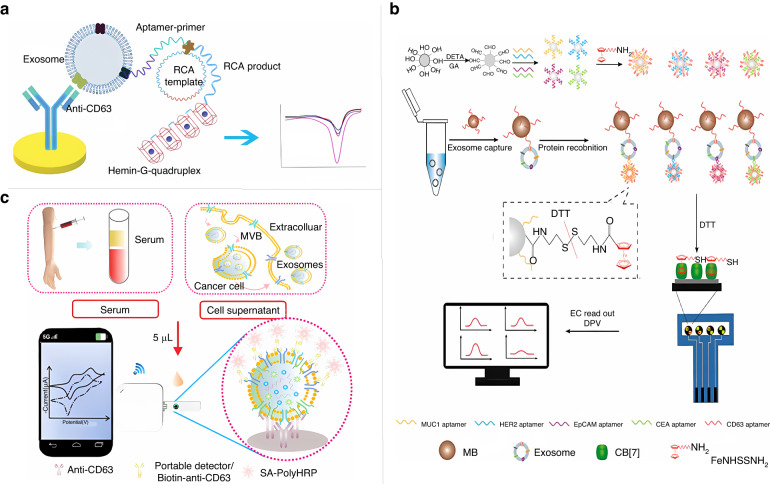
**Electrochemical sensors for exosome detection.** **a** Hemin/G-quadruplex combined with RCA for label-free, highly sensitive electrochemical aptamer detection of exosomes systems^65^. Copyright 2019 John Wiley and Sons. **b** Host-guest recognition-based magnetically mediated electrochemical sensor for simultaneous analysis of multiple breast cancer exosomes proteins^67^. Copyright 2020 American Chemical Society. **c** Smartphone combined with miniature electrochemical detector with electrode cartridge for sensitive and portable detection of exosomes in serum^70^. Copyright 2022 American Chemical Society

Exosomes are easily accessible and noninvasive biomarkers for cancer diagnosis and prognosis evaluation that can be obtained without causing any physical trauma^61^. Isolation and enrichment are required prior to exosome analysis, and traditional separation methods include ultracentrifugation and coprecipitation. These operations require expensive equipment and a large number of samples, thus limiting the use of exosomes in clinical diagnostics^62^. Among the many methods that have been developed for exosome electrochemical sensing, most use anti-CD63 antibodies or CD63 aptamers as detection probes^63^. Au et al. immobilized a CD63 aptamer on a glassy carbon electrode to capture CD63 on the exposed exosome surface and used HCR for signal amplification^64^. Huang et al. reported a label-free detection system for exosomes by combining a hemin/G-quadruplex system with RCA^65^. As shown in Fig. 4a, this detection system selects a specific gastric cancer exosome aptamer as the detection probe, and anti-CD63 modified on the electrode surface is used as the capture probe. Only this specific gastric cancer exosome captured can trigger RCA to produce multiple G-quadruplex units, and then the product is incubated with hemin to form hemin/G-quadruplex structures and catalyze the H_2_O_2_ system to produce an electrochemical signal. The sensor is highly selective and sensitive for gastric cancer exosomes with a detection limit of 9.54 × 10^2^ mL^−1^ and is expected to be a useful tool for the early diagnosis of gastric cancer. The CD63 protein is commonly found on most cellular exosomes, while the EpCAM protein is abundantly expressed in exosomes secreted by human breast cancer cells. Zhao et al. exploited this feature and used CD63 aptamer and EpCAM aptamer as capture and recognition probes for exosomes^66^, respectively; successful recognition of the exosomes triggered 3D DNA walking, and the addition of nucleic acid exonuclease III (Exo III) enabled dual-signal amplification analysis. Breast cancer is a heterogeneous disease and lacks specific tumor markers. Exosomes carry a variety of tumor-specific proteins on their surface, such as MUC1, which is highly aberrantly expressed in breast cancer; human epidermal growth factor receptor-2, an important predictive and prognostic marker of breast cancer; EpCAM, which is highly expressed in almost all adenocarcinomas; and carcinoembryonic antigen, one of the independent prognostic indicators of breast cancer. Therefore, achieving the highly sensitive detection of breast cancer using a single marker remains challenging. To address this problem, An et al. simultaneously utilized the above four proteins in combination with the CD63 aptamer and designed a sandwich structure of magnetically mediated CD63 aptamer-exosome-exosome protein to simultaneously electrochemically analyze the four markers on the surface of breast cancer exosomes (Fig. 4b)^67^. The sensor detected 1.0 × 10^7^ particles/μL exosomes in the serum of breast cancer patients with a relative standard deviation of less than 6.3%, showing great potential for the clinical diagnosis of breast cancer. Metal-organic frameworks (MOFs) have been widely used in sensor design as novel sensing materials due to their high surface area and persistent sensing properties. The high porosity of MOFs enables them to provide a large number of electroactive molecules and enhances their electrical conductivity. Wang et al. proposed a simple and effective postsynthetic modification of Zn^2+^ endogenous strategy to enhance the ECL emission of nonmetallic porphyrin-based MOF nanoluminescent clusters^68^. The material was used to construct electrochemical sensors capable of detecting 9.08 × 10^3^ particles/μL exosomes without additional identification and amplification. Although the above methods for exosome detection have yielded good results, these traditional methods are still dependent on laboratory equipment and involve complicated protocols. Therefore, it is of great importance to develop a convenient and reliable immediate detection system for point-of-care testing (POCT). Liu et al. developed a novel label-free, enzyme-free paper-based POCT electrochemical sensor using Zr-MOFs and CD63 aptamer as a recognition system^69^, combined with HCR for signal amplification, for the portable and sensitive quantitative analysis of cancer-derived exosomes. Su et al. developed a rapid, sensitive, portable electrochemical sensor that could be employed along with a smartphone for the quantification of exosomes^70^. As shown in Fig. 4c, CD63 antibody was immobilized on a screen-printed electrode to capture exosomes from human serum, and then the captured exosomes were combined with multiple biotinylated detection antibodies to form a “sandwich” complex with the exosomes. The introduced horseradish peroxidase binds to the electrode surface via streptavidin and biotin to output an electrocatalytic signal. The assay system can detect CD63-positive exosomes in 5 µL of serum in less than 2 h with a detection limit as low as 7.23 ng. As a novel electrochemical biosensing platform for the rapid in situ detection of exosomes, this sensor is a potential tool that could assist in POCT disease diagnosis, reveal new biomarkers, and support anticancer drug screening.

## Fluorescence sensing

Fluorescence sensing is one of the major detection methods in biological and chemical sciences. Fluorescence can serve as an excellent sensing probe acting as an effective transducer for transmitting fluorescent signals in biometric events collected using various detectors. Fluorescence systems incorporating nanomaterials that translate interactions between molecular recognition elements and targets into changes in fluorescence wavelength and intensity, such as fluorescence resonance energy transfer (FRET) and metal-enhanced fluorescence (MEF) effects, have been greatly improved with the continuous development of nanomaterials^71–73^.

### Fluorescence detection of tumor cell biomarkers

Fluorescence-based assays for the analysis of tumor cell biomarkers are more responsive than electrochemical assays^74^. In addition to the detection of extracellular tumor markers in body fluids using fluorescent biosensors, the in situ detection of intracellular tumor biomarkers in living cells by combining fluorescence analysis with nanodevices is also gaining attention^75^. The following section summarizes the analytical methods for fluorescence detection of intra- and extracellular tumor biomarkers in recent years.

#### Extracellular detection

##### Circulating tumor cells

**Fig. 5:**
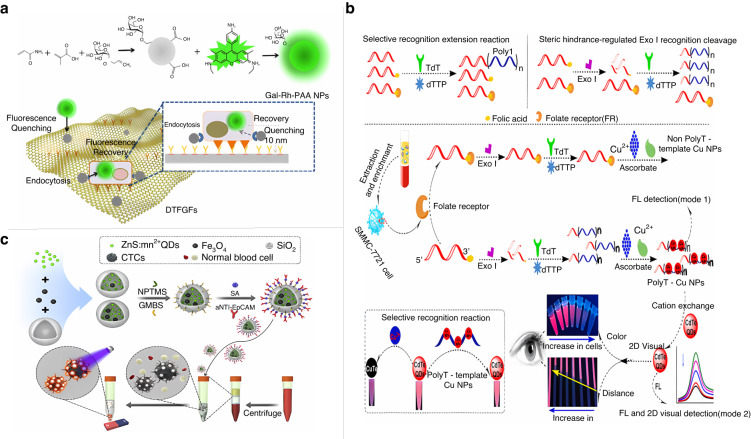
**Fluorescent biosensor for detection of CTCs.** **a** Dual-targeting functionalized reduced graphene oxide film based on EpCAM and HCC cell-specific asialoglycoprotein receptor for specific recognition of HCC-CTCs^78^. Copyright 2019 American Chemical Society. **b** Immunonanocomposites based on permanent fluorescence and magnetic properties for simultaneous capture and identification of CTCs^82^. Copyright 2019 Elsevier. **c** Dual-enzyme-assisted amplification strategy for homogeneous fluorescence as well as two-dimensional visual detection of CTCs^84^. Copyright 2022 Elsevier

In various cancers, a wealth of information on primary tumor detection, disease progression, and prognostic follow-up can be obtained by detecting CTCs in peripheral blood^76^. The high recurrence and metastasis rates of hepatocellular carcinoma (HCC) have led to extensive research on early diagnostic methods for HCC-CTCs^77^. Wu et al. created a dual-targeted functionalized reduced graphene oxide film for HCC-CTC detection^78^. As shown in Fig. 5a, the film consists of an anti-EpCAM antibody-modified graphene film and galactose-rhodamine-polyacrylamide nanoparticles. After the effective capture of HCC-CTCs, the fluorescence quenched by the rGO membrane was restored. Due to the accuracy of dual targeting, the decreased number of processing steps, and the high resolution of fluorescence imaging, this strategy can detect as few as 5 HCC-CTCs in 1 mL blood samples. Xia et al. synthesized a dual-targeted magnetic fluorescent nanobead for the simultaneous isolation and identification of HCC-CTCs;^79^ this nanobead consists of the antibody EpCAM and a small molecule near-infrared fluorescent agent with high affinity for aminopeptidase N, which is overexpressed by tumor cells. EpCAM is routinely used as a biomarker to ensure the binding and enrichment of HCC-CTCs. In addition, the aminopeptidase N-driven fluorophore not only improves the labeling efficiency of HCC-CTCs but also increases the detection purity with relatively high resolution. Therefore, using overexpressed EpCAM and aminopeptidase N as two specific targets can greatly improve the capture efficiency and detection purity of HCC-CTCs and avoid false-positive signal interference caused by the use of a single target. With the development of nanomaterials, quantum dots (QDs) have been widely used in the field of fluorescence detection due to their broad excitation and narrow emission characteristics^80,81^. As shown in Fig. 5b, Cui et al. encapsulated ZnS:Mn^2+^ QDs and Fe_3_O_4_ nanoparticles into SiO_2_ nanospheres and then modified the surface of the nanospheres with anti-EpCAM antibodies to form multifunctional immunomagnetic nanomaterials^82^. After capturing CTCs from patients’ blood and performing magnetic separation, CTCs could be directly identified by yellow‒orange light emitted from ZnS:Mn^2+^ QDs under a fluorescence microscope. The strategy successfully achieved a high capture efficiency of up to 90.8% and achieved specific fluorescent labeling of CTCs in samples from nine clinical breast cancer patients. Chen et al. reported a simple all-nucleic acid enzyme-free amplification strategy for the fluorescence detection of lung cancer cells in clinical blood samples with CdTe QDs capable of selectively recognizing released Ag^+^ to generate visual and fluorescent signals as nanoscale signal reporters, using MUC1 as a CTC marker and aptamers as recognition probes^83^. Under optimized conditions, the detection of MUC1 down to 0.15 fg/mL was achieved with a fluorescent signal. MUC1 at 1 fg/mL could be detected with the naked eye. The ability of this strategy to generate visual signals without instruments is expected to provide a solution for clinical diagnosis in POCT. Thereafter, the group used the CTC marker folate receptor to selectively bind to the probe folate-T30 (Fig. 5c)^84^. After binding, folate-T30 cannot be recognized and cleaved by exonuclease I, inhibits subsequent terminal deoxynucleotidyl transferase recognition of the substrate, and catalyzes the extension reaction. The CdTe QDs would not be able to recognize the generated polyT-CuNPs and free Cu^2+^ to generate the corresponding fluorescent signals. The strategy can also be combined with inkjet printing technology for remote visualization and reading on the basis of instrument-free visual detection, and its fluorescence signal detection limit is as low as 0.25 cells/mL, and the signal can be distinguished by the naked eye at 1 cell/mL, providing a potential solution for current issues in POCT of CTCs. In addition to the enrichment and capture of CTCs using biochemical methods, Song et al. invented the physical method of microfluidic porous membrane filtration to efficiently capture CTCs stained by fluorescence^85^. This invention is an important guideline for the development of a microfluidic porous membrane automatic filtration separation system for CTCs and for the improvement of the capture efficiency of CTCs.

##### Circulating tumor DNA

**Fig. 6:**
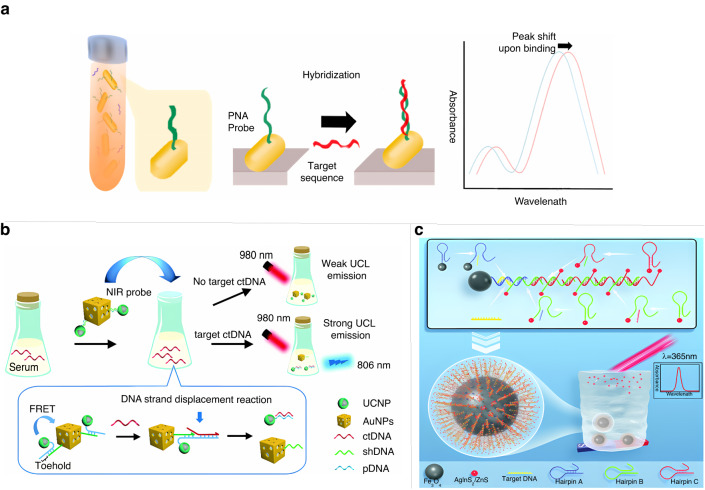
**Fluorescent biosensor for ctDNA detection.** **a** Amplification-free fluorescence detection of ctDNA using peptide nucleic acid probes with gold nanorods^89^. Copyright 2019 Elsevier. **b** Upconversion nanoparticle-based FRET system for ctDNA detection^95^. Copyright 2020 Royal Society of Chemistry. **c** Fluorescent sensor for ctDNA detection based on HCR-mediated accumulation of AgInS2/ZnS QDs^97^. Copyright 2022 Royal Society of Chemistry

Due to the low concentration of ctDNA, both the sensitivity and specificity of the sensor are critical, and conventional detection methods are time-consuming and costly^86^. When light is incident on nanoparticles composed of precious metals, if the incident photon frequency matches the overall vibration frequency of the precious metal nanoparticles or metal conduction electrons, the nanoparticles or metal will have a strong absorption effect on the photon energy, which results in the phenomenon of local surface plasmon resonance; this can be seen as a strong resonant absorption peak on the spectrum^87^. This principle can be used for the direct label-free and highly sensitive detection of ctDNA^88^. Tadimety et al. used a peptide nucleic acid probe coupled to a gold nanorod for sequence-specific capture of ctDNA in solution, and the capture of ctDNA resulted in a change in the wavelength of the absorption peak of the nanorod (Fig. 6a)^89^. This sensor provides a new method for the amplification-free fluorescence detection of ctDNA with an effective detection limit of 2 ng/mL in patient serum. In recent years, nanomaterials have been widely used in the construction of novel biosensing platforms due to their excellent optical and electrocatalytic properties. For example, graphene oxide^90^ and molybdenum disulfide^91^ have been used as efficient nanobursting agents to develop various fluorescent biosensors. However, the fluorescence bursting ability of similar nanomaterials is relatively inefficient, which limits their wide practical applications. Therefore, new homogeneous 2D materials^92^ with good fluorescence bursting efficiency have attracted attention. Zhang et al. used homogeneous 2D palladium nanomaterials combined with a pair of DNA detection probes with single-stranded sticky ends^93^. The high fluorescence burst efficiency and differential affinity of single/double-stranded DNA by the palladium nanomaterials enabled the detection of ctDNA without signal amplification with a detection limit of 0.63 nM. Huang et al. prepared 2D nitrophenyl-functionalized black phosphorus nanosheets (NP-BPs) for the selective detection of ctDNA, and the NP-BPs showed higher burst efficiency and stronger affinity for single-stranded DNA than double-stranded DNA^94^. When the FAM-labeled single-stranded DNA probe forms double-stranded DNA in the presence of a specific ctDNA target, the burst FAM generates a fluorescent signal due to the inability of the double-stranded DNA to adsorb to the NP-BPs, and the sensor provides reliable readings within 15 min. Although the detection results of organic dye-based fluorescence techniques are satisfactory, the background fluorescence and self-absorption phenomena of biological samples severely limit the clinical application of fluorescence-based molecular diagnostic techniques. Therefore, the development of new fluorescence systems for the clinical detection of ctDNA is urgently needed. Wang et al. constructed a FRET-based system for the sensitive detection of ctDNA, which takes advantage of upconversion nanoparticles (UCNPs) that can be excited by near-infrared light, are chemically stable, do not fluoresce in biological tissues themselves, and cause little damage to biological tissues by assembling two partially complementary single-stranded DNA segments with gold nanocages (AuNCs) based on their surface modifications (Fig. 6b)^95^. The high extinction coefficient of AuNCs leads to the bursting of upconversion luminescence. When the foothold region of single-stranded DNA on UCNPs is used to capture free ctDNA in the sample, the ctDNA triggers a foothold-mediated strand replacement reaction and then separates from the AuNCs, and the fluorescence of UCNPs is recovered. Blank spiked recovery experiments were performed using this sensor for ctDNA in human serum samples, and the recovery was in the range of 93.14% to 98.51%. Among the many nanomaterials applied for fluorescence detection, the unique physical properties of QDs have received much attention from researchers, but the commonly used QDs have a certain degree of toxicity, and the study of fluorescence detection based on nontoxic QDs is particularly important^96^. AgInS_2_ QDs, which have been involved in significant research progress in recent years, have not only the excellent properties of QDs but also the advantages of low toxicity and environmental friendliness. Yang et al. designed a sensor for the accumulation of AgInS_2_/ZnS QDs via ctDNA-triggered HCR, which triggers a significant change in the fluorescence signal^97^. As shown in Fig. 6c, the target DNA triggers the HCR reaction for alternate hybridization between hairpin probes B and C, resulting in the accumulation of QDs attached to B and C. Since hairpin probe A, which can specifically capture ctDNA, is modified on the magnetic nanoparticle Fe_3_O_4_, ctDNA can be quantified by detecting the intensity of the fluorescence signal produced by QDs in the supernatant after magnetic separation. The HCR process leads to the effective accumulation of QDs, indicated by luminescence, which amplifies the detection signal and improves the detection sensitivity with a detection limit as low as 53 aM, and this study provides a rapid, convenient, and sensitive method for ctDNA detection.

##### Exosomes

**Fig. 7:**
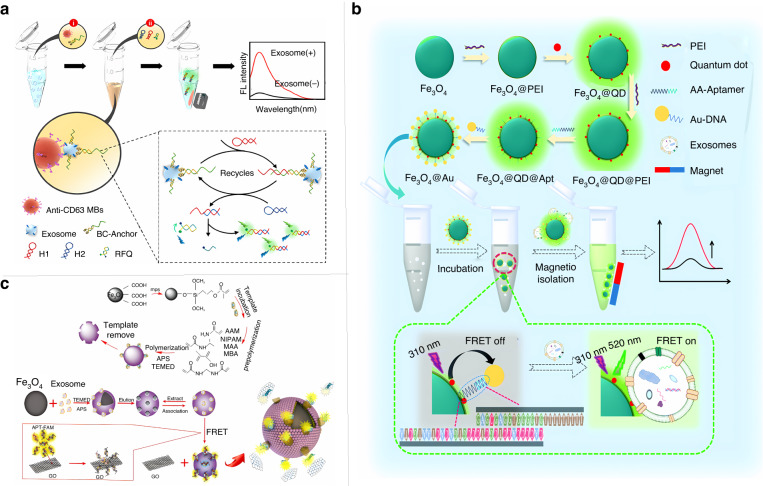
**Fluorescent biosensor for exosome detection.** **a** Immunomagnetic bead-based capture and separation method for fluorescence analysis of exosomes^99^. Copyright 2021 American Chemical Society. **b** Nucleic acid aptamer-functionalized FRET magnetic nanoparticle system for exosome detection^101^. Copyright 2021 Royal Society of Chemistry. **c** A novel dual-selective FERT-based fluorescent sensor for exosomes of MIPs^102^. Copyright 2022 Elsevier

Magnetic nanomaterials are widely used for the detection of exosomes because they can effectively remove impurities and excess reagents. Wu et al. proposed a magnetic and fluorescent biosensor constructed by using a CD63 aptamer to modify magnetic microspheres loaded with a large number of QDs via a DNA probe for exosome recognition^98^. After the aptamer captures exosomes, the QD-DNA probe is detached from the magnetic microspheres to generate a fluorescent signal. Wang et al. used anti-CD63 antibody-immobilized magnetic beads to capture and enrich exosomes from complex samples and then introduced bivalent cholesterol sequences into the lipid bilayer membrane of the captured exosomes after performing enrichment by exploiting hydrophobic interactions between cholesterol and the lipid bilayer^99^. As shown in Fig. 7a, loop amplification of enzyme-free DNA signal amplification is triggered by the action of a hairpin probe, and probes containing the fluorophore and bursting agent are introduced during loop amplification to detect and analyze exosomes. Under optimal conditions, exosome detection was achieved in the range of 5.5 × 10^3^ to 1.1 × 10^7^ particles/mL, with a detection limit of 1.29 × 10^3^ particles/mL. In addition to fluorescence detection based on magnetic nanomaterials, the FRET-based fluorescence analysis system provides a new platform for the quantitative analysis of various biomolecules by simultaneously detecting the signals of donors and acceptors. According to Xiong et al., based on proximity hybridization-mediated aptamer-specific recognition and FRET, non-small cell lung cancer exosomes coexpressing CD63/EGFR/EpCAM were efficiently captured^100^. Zhu et al. established a nucleic acid aptamer-functionalized FRET magnetic nanoparticle system by combining nucleic acid aptamer-mediated exosome detection and the FRET effect^101^. As shown in Fig. 7b, this system introduces QDs and aptamers onto magnetic nanoparticles, and when the aptamers are paired with complementary DNA on the surface of gold nanoparticles, the formed QDs-Apt/DNA-Au complexes display weak fluorescent signals due to the FRET OFF mechanism. When the corresponding exosomes bind to the aptamer and decompose Au-DNA, the fluorescence signal is enhanced by the FRET ON mechanism. The constructed FRET magnetic aptamer sensor was able to distinguish 7 samples of lung cancer and 5 samples of other cancers from healthy control samples quickly and effectively with 100% accuracy. The use of antibodies specific to exosome surface proteins for recognition is a strategy that has yielded good results but is still limited by high antibody cost, instability, batch variation, and antibody unavailability. Molecularly imprinted polymers (MIPs), synthetic receptors prepared by blotting polymerization using target molecules as templates, have been widely used as an alternative to antibodies for biosensing analysis because of their stability, simplicity of preparation, and high affinity and selectivity for receptors. Feng et al. constructed a novel dual-selective FERT-based fluorescent sensor for exosomes of MIPs^102^. As shown in Fig. 7c, the sensor uses magnetic MIPs to selectively trap exosomes and form a sandwich structure with aptamer/graphene oxide to cause the selective “turn-on” of FRET with a detection limit of 2.43 × 10^6^ particles/mL for exosomes in serum. The unique ability of MIPs to capture and detect incomplete or unknown targets provides a new idea for the clinical detection of certain targets, such as tumor cells, with incomplete information. Chen et al. used a laser emitter to make the AptCD63 magnetic bead-exosome complex in the detection chamber emit fluorescence; when this fluorescence was received, the light signal could be converted into a voltage signal and output to a computer according to the light intensity of this fluorescence to obtain the concentration of exosomes^103^. This POCT device has laid a solid foundation for effectively solving the problems of expensive traditional optical testing instruments.

#### Intracellular detection

##### Telomerase

**Fig. 8:**
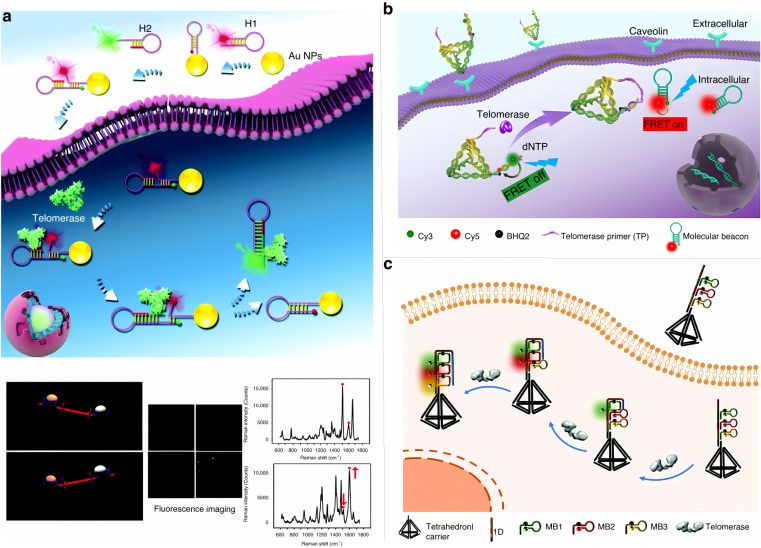
Fluorescence analysis of telomerase in living cells. **a** Dual-signal analysis of telomerase based on fluorescence and surface-enhanced Raman scattering^105^. Copyright 2019 Royal Society of Chemistry. **b** Tetrahedral DNA nanoprobe based on FRET function for telomerase detection^108^. Copyright 2020 Elsevier. **c** A transfectant-free DNA tetrahedral probe for real-time monitoring of telomerase activity^109^. Copyright 2021 Royal Society of Chemistry

Overexpression of telomerase in tumor cells leads to the unlimited proliferation of cancer cells^104^. The development of sensitive probes for fluorescent imaging and detection of low-abundance telomerase activity in living cells poses a great challenge, namely, the need to deliver the probe into the cell and complete detection while preserving cellular activity. Fan et al. designed a nanoprobe based on a cascade amplification reaction, which was constructed by the physisorption of a DNA enzyme and a catalytic hairpin probe bound to a MnO_2_ nanosheet^26^. Once the probe was endocytosed by tumor cells, the MnO_2_ nanosheets were degraded and released all DNA strands, thereby recognizing telomerase and leading to recovery of the probe’s fluorescence. The probe was successfully used to monitor the dynamics of telomerase activity in cervical cancer cells as well as in three other cell types. Ye et al. designed a seesaw ratiometric (SR) probe combining fluorescence and surface-enhanced Raman scattering (SERS) techniques (Fig. 8a)^105^. The SR probe was synthesized by the hybridization of two hairpin DNAs, H1 modified with nanogold and Cy3 dye at opposite ends and H2 modified with Rox dye and containing telomerase primer. In the absence of telomerase, the Rox dye is close to the nanogold, and the Cy3 dye is far from the nanogold, which results in a fluorescence off/Raman on phenotype for Rox and a fluorescence on/Raman off phenotype for Cy3. When the SR probe is taken up by the tumor cell through endocytosis of the cell membrane, a competitive hybridization reaction between H1 and H2 is triggered in the presence of telomerase, leading to the dissociation of the SR probe and the release of H2. The hairpin structure of H1 and H2 is restored, leading to a fluorescence on/Raman off phenotype for Rox and a fluorescence on/Raman off phenotype for Cy3 in the SR probe. This strategy detects 8.604 × 10^−12^ IU of telomerase activity in a single live breast cancer cell using dual-signal reverse change imaging, providing unprecedentedly reliable imaging information for telomerase detection. In addition to conventional DNA probes, tetrahedral DNA nanostructures have been used as nanocarriers to deliver nucleic acids into cells due to their good stability and permeability^106,107^. Yue et al. designed a tetrahedral DNA nanoprobe with FRET function^108^. As shown in Fig. 8b, the probe consists of a telomerase primer and molecular beacons labeled with fluorophores Cy3 and Cy5, where the telomerase primer is extended in the presence of telomerase, and the FRET-based molecular beacon is replaced to stimulate the FRET response. This strategy allows tetrahedral DNA nanoprobes to deliver nucleic acids into the cytoplasm for detection via endocytosis without the use of transfection reagents. Zhang et al. designed a DNA tetrahedral probe that can be directly endocytosed into cells without transfection agents^109^. As shown in Fig. 8c, the DNA tetrahedral probe can be loaded with telomerase primers at the tip as well as molecular beacons labeled with three different fluorophores. In the presence of telomerase, the molecular beacons loaded on this probe are turned on continuously, sequentially generating multicolor fluorescence signals with different emission wavelengths. The good biocompatibility of this DNA tetrahedral probe provides a new idea for the real-time monitoring of telomerase activity in tumor cells. Wang et al. combined two techniques, rolling loop amplification and catalytic hairpin amplification, with microfluidic microarray technology for in situ fluorescence imaging of telomerase in living cells^110^. This method enables the simultaneous monitoring of telomerase activity and its changes in different tumor cells within 1.5 h with only 1–2 μL of cell suspension and reagents. However, this multiple artificial amplification-based strategy is likely to cause serious interference with the results of in situ monitoring. To directly analyze telomerase activity in vivo and thereby better understand tumor progression, Dai et al. constructed a distance-dependent magnetic resonance-tuned telomerase activation magnetic resonance imaging probe for direct analysis of telomerase activity in vivo^111^. This probe provides a directly readable output signal and enables the in situ screening of telomerase inhibitors in a whole animal model, providing an additional tool for early tumor diagnosis and assessment of the therapeutic process. Nevertheless, the susceptibility of telomerase primers and signal-amplified DNA components to degradation by intracellular nucleases upon delivery into cells, the nonspecific assembly of DNA units that may result in false signals, and the cost of labeling DNA probes for in situ telomerase monitoring systems are still challenges that many researchers are currently facing.

##### miRNA

**Fig. 9:**
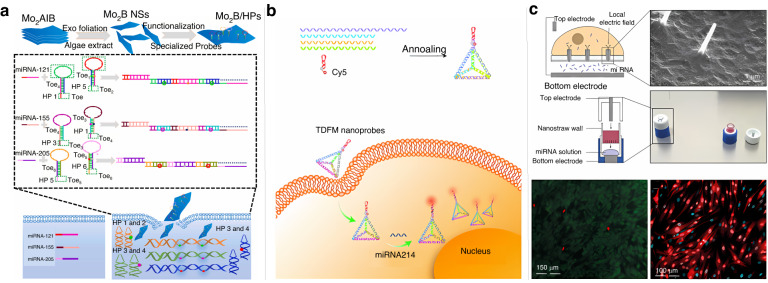
**Fluorescence analysis of miRNA in living cells.** **a** Live cell multiplexed amplified miRNA monitoring based on Mo2 B fluorescence burst and HCR^114^. Copyright 2021 Elsevier. **b** FRET-based functional tetrahedral DNA nanoprobe for telomerase detection^120^. Copyright 2022 Royal Society of Chemistry. **c** A transfectant-free DNA tetrahedral probe for real-time monitoring of telomerase activity^123^. Copyright 2021, John Wiley and Sons

miRNAs are expressed in specific tissues and cell types and are expressed dynamically with different developmental or disease stages^112^. Due to low expression levels and complex environments, the sensitive detection of miRNAs in living cells requires multifunctional vectors with high transfection efficiency. Lu et al. used miRNA mimic-containing cell membranes encapsulated with both modified gold nanoparticles and double-stranded specific nucleases as nanocarriers to successfully detect oncogenic miRNAs in MCF-7 cells and showed that this system can be used to monitor dynamic changes in oncogenic miRNA expression in cancer cells^113^. Zada et al. were the first to use a 2D Mo_2_B nanosheet as a carrier and constructed a simple and sensitive multi-miRNA imaging platform, exploiting its ability to quench fluorescent dyes in combination with HCR to successfully monitor the changes in miRNA expression in cancer cells^114^. As shown in Fig. 9a, the large surface of the Mo_2_B nanosheet allowed the loading of more hairpin probes, and a fluorescence burst from the hairpin probes was observed. After transfection, the hairpin probes recognize specific target miRNAs, and the HCR reaction is triggered to produce long DNA‒miRNA double helix structures that dissociate from the Mo_2_B nanosheet surface and produce strong fluorescence. This method enables the imaging of multiple miRNAs in different cells to distinguish cancer cells from normal cells, providing a new way to analyze the expression patterns of miRNAs in living cells. Although various nanomaterials have been effectively used as carriers for miRNA detection, their potential toxicity and biocompatibility in biological systems cannot be ignored^115^. In particular, some nanoparticles based on metals and metal oxides may cause health problems such as inflammation, DNA damage, and even accelerated tumorigenesis in humans through various mechanisms during use. To ensure that nanomaterials can continue to play a positive role in biomedicine, it is necessary to select appropriate nanomaterials according to the characteristics and requirements of the detection system to avoid the use of nanomaterials with high toxicity at the source. In addition, it is necessary to control the concentration and dosage of nanomaterials to prevent the overuse of nanomaterials that may interfere with the test results or even expose the recipient to additional health hazards. Silicon quantum dots (Si QDs) are representative inorganic nanomaterials in biomedicine due to their negligible toxicity and superior biocompatibility^116^. Mahani et al. designed a molecular beacon based on Si QDs and Black Hole Quencher-1 for the detection of miRNA-21 in human serum via the FRET mechanism^117^. The method shows the great potential of multifunctional Si QDs for biomedical applications. Moreover, it was shown that DNA tetrahedral-based nanostructures can easily penetrate cell membranes and resist nuclease degradation in living cells. Taking advantage of this, Xing et al. designed a DNA tetrahedron-based molecular beacon as a probe to rapidly trigger a catalytic hairpin self-assembly reaction in the presence of target miRNAs to amplify the fluorescent signal to complete the detection^118^. Li et al. similarly proposed a DNA tetrahedral framework combined with a molecular beacon nanoprobe to specifically recognize miR-214, which plays an important role in tumor proliferation and metastasis (Fig. 9b)^119^. In contrast to the former, the molecular beacon modified with fluorophores and bursting agents at both ends is embedded in one apex of the DNA tetrahedral framework, and in the absence of miR-214, the nanoprobe is in the “off” state due to the FRET effect. When this nanoprobe is phagocytosed by cells, the molecular beacon recognizes miR-214, and then the probe structure changes, leading to the recovery of the fluorescent signal. This method enables real-time fluorescence imaging of tumor-associated miRNAs in living cells. Compared with DNA-based probes, chemically modified nucleic acid analog probes have stronger binding affinity and specificity for target RNAs. LO et al. invented a threose nucleic acid (TNA)-based probe for the detection and imaging of miRNA in living cells. The probe consists of a fluorophore-labeled TNA reporter strand partially hybridized with a quencher-labeled TNA recognition strand, which can quantify miRNA by fluorescence enhancement after its entry into cells without harmful transfection treatment^120^. Moreover, the TNA probe can be efficiently taken up by living cells, and its cytotoxicity is negligible. This probe can be used to monitor target miRNAs in real-time as well as to differentiate the expression levels of different target miRNAs in various cancer cell lines. In addition, DNA nanowires have been shown to enter the cytoplasm without a co-carrier agent. Wu et al. assembled DNA enzymes onto DNA nanowires, and the targets could specifically initiate the cleavage reaction of the DNA enzymes along the DNA nanowires^121^, thereby producing an enhanced fluorescent signal. miRNA can be detected by translocation of the probe into the cell through the deformation of the cell’s own plasma membrane during endocytosis. Pop et al. designed another miRNA detection method employing a nanostraw-based electroporation delivery platform^122^. As shown in Fig. 9c, this platform delivers miRNA directly into living cells through a combination of low-pressure electroporation and electrophoresis. After miRNA delivery is completed and the electric field is turned off, the cell membrane can heal rapidly, within 10 min. This strategy is expected to enable basic research probing the changes in signaling in disease states due to miRNA concentration changes with minimal interference to cells.

## Commercialized technologies for liquid biopsies

With the rapid development of biotechnology in recent years, an increasing number of researchers and companies are focusing on developing commercial liquid biopsy technologies for early tumor diagnosis and treatment. Biocept is a biotechnology company specializing in cancer detection technology. The test has demonstrated high accuracy and sensitivity in multiple clinical trials^123^. In addition, the Guardant360 blood test technology developed by Guardant Health identifies specific variants of tumors and helps physicians choose the most appropriate treatment option for their patients. Umemoto et al. used Guardant360 to perform ctDNA analysis in patients with pancreatic ductal adenocarcinoma during disease progression, thereby demonstrating the clinical utility of ctDNA analysis in pancreatic ductal adenocarcinoma with liver metastases^124^. Another commercially available option for ctDNA analysis is CancerSEEK blood test technology developed by Thrive Earlier Detection. Killock et al. mentioned that the technique can be used to diagnose eight cancer types, such as breast, colorectal, and gastric cancers, and its specificity of detection is up to 99%, which is expected to develop into a noninvasive blood test technique for multianalyte testing^125^. Illumina, a biotech company known for its whole genome sequencing technology, bases its TruSight Oncology 500 blood test primarily on next-generation gene sequencing technology^126^. Hsieh et al. Li et al. used a comprehensive panel of this test technology to process sequencing data to identify small nucleotide variants and identified pathogenic activating mutations such as PIK3CA and loss-of-function of TP53 in multiple patients^127^. Beyond the direct early detection of tumors in blood samples, AI software has a wide range of applications in detecting and diagnosing early-stage diseases. Freenome Medical Technologies combines cutting-edge technology and proven medical theories to radically reduce cancer mortality by analyzing DNA fragments in human blood samples through AI, working to detect cancer at an early stage and stop the spread of the disease^128^. Although the detection technologies developed by these biotech companies need further refinement and optimization, they can undoubtedly provide new possibilities and hope for early cancer screening and diagnosis as long as they are driven by continuous innovation and technological advances. In the future, these technologies are expected to become an important method for noninvasive cancer screening and enable more patients to be treated as early as possible through more accurate tumor detection and diagnosis.

## Summary and outlook

In this review, we summarize the detection methods for extracellular and intracellular tumor biomarkers based on two main principles: electrochemistry and fluorescence. Among the detection methods for extracellular tumor markers, electrochemical sensing shows high sensitivity and selectivity (Table 1), but its stability and reproducibility still need further study to prevent misleading results in liquid biopsies. Fluorescence sensing also shows good sensitivity and low background signal detection and has shown good promise in POCT, but its detection speed and efficiency are still less than satisfactory. The fluorescence detection of intracellular markers still faces great challenges due to the need to balance cellular activity, high accuracy, and sensitivity; for example, the toxicity of nanomaterials cannot be ignored when using endocytosis to deliver nanoprobes for detection. A good understanding of the toxicity of nanomaterials will help to design nanomaterials with fewer side effects, and the development of mechanisms to assess the toxicity of nanomaterials in vivo and ex vivo is an important part of achieving in situ monitoring of tumor cells. Moreover, the integration of various safe nanoprobes into wearable ultrasensitive biosensors and the translation of existing technologies from the laboratory to clinical practice remain major challenges in achieving early monitoring of tumor markers. With the emergence and continuous improvement of physical delivery methods such as electroporation, future research should focus on building standardized nanodevices to improve the efficiency of early and precise clinical detection and targeted treatment of tumor cells and thus meet the needs of large-scale commercial applications.

**Table 1:** Limit of detection (LOD) of each tumor biomarker by different biosensing techniques

Sensing Technique	Target	Theory	LOD	Ref.
Electrochemical	CTCs	Dual antibody recognition	1 cell/mL	^46^
	ctDNA	DNAzyme ratio assay	25 aM	^54^
	exosomes	MOFs ECL assay	9.08 × 10^3^ particles/μL	^68^
Fluorescence	CTCTs	CdTe QDs visual detection	0.25 cells/mL	^84^
	ctDNS	AgInS2/ZnS QDs & HCR amplification	53 aM	^97^
	exosomes	Dual-selective MIP recognition	2.43 × 10^6^ particles/L	^102^
	telomerase	In situ fluorescence imaging	1LoVo cell	^110^
	miRNA	TDFM nanoprobe recognition	5 nM	^119^
